# Dataset of herbarium specimens of threatened vascular plants in Catalonia

**DOI:** 10.3897/phytokeys.77.11542

**Published:** 2017-02-23

**Authors:** Neus Nualart, Neus Ibáñez, Pere Luque, Joan Pedrol, Lluís Vilar, Roser Guàrdia

**Affiliations:** 1 Institut Botànic de Barcelona (IBB-CSIC-ICUB), Passeig del Migdia s/n, E-08038 Barcelona, Spain; 2 Museu de les Terres de l’Ebre, c/ Gran Capità 34, E-43870 Amposta, Spain; 3 Institut d’Estudis Ilerdencs, Plaça Catedral s/n, E-25002 Lleida, Spain; 4 Universitat de Girona, Campus de Montilivi, E-17071 Girona, Spain; 5 CeDocBiV CRAI Universitat de Barcelona, Baldiri Reixac 2, E-08028 Barcelona, Spain

**Keywords:** Catalonia, conservation, Cormophyta, herbarium, northeastern Iberian Peninsula, specimen, threatened flora

## Abstract

This data paper describes a specimens’ dataset of the Catalonian threatened vascular plants conserved in five public Catalonian herbaria (BC, BCN, HGI, HBIL and MTTE). Catalonia is an administrative region of Spain that includes large autochthon plants diversity and 199 taxa with IUCN threatened categories (EX, EW, RE, CR, EN and VU). This dataset includes 1,618 records collected from 17^th^ century to nowadays. For each specimen, the species name, locality indication, collection date, collector, ecology and revision label are recorded. More than 94% of the taxa are represented in the herbaria, which evidence the paper of the botanical collections as an essential source of occurrence data.

## Introduction

For the maintenance of ecosystem processes, effective conservation is essential (Rands et al. 2010) and natural history collections have been recognized as a valuable source of data for applied these conservation efforts (Krupnick et al. 2009) due they are permanent and well-documented distribution records of taxa through time and space. There are many studies that show how the specimens stored in herbaria are useful for better knowledge of endangered flora; for instance, to evaluate the impact of over-collecting in the past for nowadays extinct plants (Aedo et al. 2015), to prioritize regionally rare plants for conservation (Kricsfalusy and Trevisan 2014) or to evaluate threatened flora hotspots (Mendoza-Férnandez et al. 2015). But not only the primary data included in the label is useful for biodiversity studies, Greve et al. (2016) shows how other information related to the specimen’s environment can provide distribution maps of soils types or vegetation; and Calinger et al. (2013) demonstrate changes on the flowering phenology due to climate change from the visual examination of the specimen’s flowers.

## Project description

### Purpose

The aim of this project is (1) to join the specimens’ data of endangered plants in Catalonia in a unique dataset, (2) to improve the accessibility of this data for conservation purposes, (3) to describe the taxonomical, chorological and temporal diversity of this dataset and (4) to evaluate if it is representative of this kind of flora. Five public herbaria have participated and all their data have been published through GBIF in a unique dataset. The herbaria included are those who already have these specimens informatizated but in the future we plan to include more collections.

Some of these herbaria have yet evaluated their specimens of threatened plants in previews works. In the herbarium of the Botanic Institute of Barcelona (BC) the specimens of some collections have been analyzed to assess if the threatened but also the endemic taxa of Catalonia were well represented in the herbarium (Nualart et al. 2012). Results showed that specimens from Catalonia conserved in BC represented 82.24% of the 304 endemic and threatened taxa, a high percentage that demonstrates that this herbarium has a good representation of this flora. We expect that the representation of threatened flora in the present project would grow significantly as the number of collections studied increases.

### Study area

**Figure 1.**
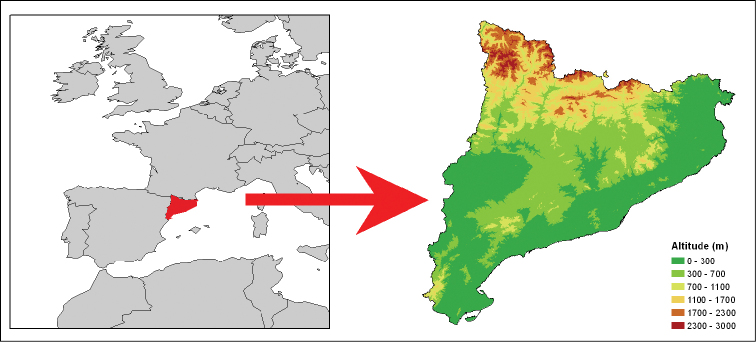
Location of Catalonia and elevation map of the study area.

Catalonia is an administrative region in the northeastern corner of Spain in the Mediterranean Coast that covers approximately 32,000 km² (Figure 1). It includes a large biogeographic, physiographic and orographic diversity due to the presence of the Pyrenees in the north and the Mediterranean Sea in the east. The most abundant climate is Mediterranean, characterized by warm winters and hot and drought summers. The annual average temperature ranges between 1°C above 2,000 m in the Pyrenees and 18°C below 50 m. Annual precipitation ranges from 200 mm in the Catalan Central Depression to more than 1,250 mm in some areas of the Pyrenees.

The vascular flora of Catalonia includes 4,831 taxa (Font 2016) that constitute a relevant part of the Western Mediterranean flora. There are plants from three principal biogeographic regions (Bolòs and Vigo 1984): (a) the Mediterranean flora, characterized by sclerophyllous forests and shrubs, (b) the Euro-Siberian (including Atlantic and Sub-Mediterranean) elements that reach the rainy mountains and are characterized by deciduous forests and mesic grasslands, and (c) the Boreo-Alpine elements of the mountains highlands, with subalpine forests and alpine grasslands.

### Design description: threatened taxa

In this project, we have compiled the records of the threatened taxa specimens included in the Catalonia Red List (Sáez et al. 2010: 772). This Red List includes species and subspecies which are in a higher or lower risk of extinction, and those that have become extinct nowadays. It includes 199 threatened taxa according to the IUCN categories (IUCN 2012). The 45.72% are vulnerable (VU), with a high risk of endangerment, the 27.13% are endangered (EN), with a high risk of extinction and the 18.59% are critically endangered (CR), with an extremely high risk of extinction. The rest are extinct, either regionally or globally; the 8.04% are locally extinct in this region (RE)—although one (*Marsilea quadrifolia*) is only extinct in the wild thanks to a cultivated population in the area (RE (EW))—and finally there is an endemic taxon considered totally extinct (EX), *Festuca paucispicula*.

### Design description: herbaria

This project includes the specimens conserved in the following public herbaria of Catalonia: (1) BC of the Botanical Institute of Barcelona, (2) BCN of the University of Barcelona, (3) HGI of the University of Girona, (4) HBIL of the Public Foundation *Institut d’Estudis Ilerdencs* and (5) MTTE of the museum *Museu de les Terres de l’Ebre*. The BC herbarium (http://www.ibb.bcn-csic.es/en/documentacio/herbari/) is the largest collection of plants in Catalonia, and the second in Spain. This collection, with about 800,000 specimens, is specialized in western Mediterranean flora and has served as reference for the main floras of this region. In this project we have included the general collection and also historical collections as the Salvador’s herbarium from 17_th_ and 18_th_ century, the collections of F. Trèmols (1831–1900) and E. Vayreda (1848–1901) from 19_th_ century and those of J. Cadevall (1846–1921) and Fr. Sennen (1861–1937) from 19_th_ to early 20_th_ century, which allows a very high temporal representation. The BCN herbarium (http://crai.ub.edu/ca/coneix-el-crai/CeDocBiV/herbari) hosts more than 400,000 specimens, with a great representation of all the major groups of plants. This herbarium has a wide range of specimens from the Pyrenees, the Ebre Basin, the Mediterranean coastal areas and some tropical South American. As for historical collections, the most outstanding are those of J. Planellas (1821–1888), J. Teixidor (1836–1885), Fr. Sennen, P. Font Quer (1888–1964) and T.M. Losa (1893–1965). HGI herbarium, created in 1976, stores about 23,200 specimens mainly of the Girona province and also includes a historical collection of Isern’s herbarium from 19_th_ century, and the collection of L. Pericot (1899–1978). The HBIL herbarium was created in 1942 and stores more than 15,000 sheets of vascular plants collected basically from the Lleida province. The MTTE herbarium includes near 3,500 specimens of the Ebre delta regional area.

Although some of these specimens are already available in GBIF portal (CeDoc de Biodiversitat Vegetal: BCN-Cormophyta http://www.gbif.org/dataset/834f1756-f762-11e1-a439-00145eb45e9a; Institut Botanic de Barcelona, BC http://www.gbif.org/dataset/838475f4-f762-11e1-a439-00145eb45e9a; Universitat de Girona: HGI-Cormophyta http://www.gbif.org/dataset/835727b6-f762-11e1-a439-00145eb45e9a), this new dataset includes specimens not yet published and summarizes the data from all these herbaria. The information provides a joint and overall vision of all the specimens that will be useful for conservation policy and scientific research.

### Data published through GBIF

http://www.gbif.es/ipt/resource?r=threatenedcat

## Herbarium coverage

The searching of endangered plants in the herbaria allowed us to find 1,618 specimens from Catalonia. Although in these herbaria there are also many specimens collected in other Spanish regions or other countries, we have only recorded those collected in Catalonia. Table 1 shows the number of taxa and the specimens founded for each IUCN categories. More than 94% of the 199 taxa are represented in the herbaria, which evidences the paper of the botanical collections as an essential source of occurrence data. Only the regionally extinct taxa (RE) are less present in the herbaria due to the old bibliographic cites without voucher specimens. However, the 96.70% of the endangered taxa (VU, EN and CR) have almost one specimen, which demonstrates the good representativeness of this dataset per this kind of flora.

**Table 1.** Representation of the Catalonian threatened taxa in the dataset.

	**Taxa**			**Specimens**
	**Red List**	**Herbarium**	%	
**VU**	91	90	98.90	927
**EN**	54	53	98.15	455
**CR**	37	33	89.19	182
**RE + RE (EW)**	16	11	68.75	48
**EX**	1	1	100.00	6
**TOTAL**	199	188	94.47	1,618

Table 2 shows the number of taxa according to the number of specimens stored in the herbaria and the IUCN category. The taxa with more than 30 specimens only represent the 4.26% of all the taxa included in the dataset, while more than 50% of the taxa have a few number of them (20 taxa only with a single specimen). We can see a general trend of the number of taxa decline as the number of specimens increases (from left to right of the table), and another general trend of the number of specimens decline as the threat degree increases (from top to down of the table), already observed in Nualart et al. (2012). These trends evidence that taxa with lower risk of extinction are more collected than the more endangered ones. This fact is due because usually these last taxa are infrequent, often with a small population size, a restricted and/or fragmented distribution area and come from difficult access habitats. Furthermore, many of these taxa have some type of protection that prohibits their gathering according to the Catalogue of Endangered Flora (DOGC 2008; 2015). These characteristics could explain the difficulty in their gathering and therefore, their small number of specimens.

**Table 2.** Number of taxa according to the specimens’ range and the IUCN category.

	Number of specimens						
	1–5	6–10	11–15	16–20	20–25	25–30	> 30
**VU**	43	19	12	5	2	2	7
**EN**	27	11	6	7	1	.	1
**CR**	20	8	3	2	.	.	·
**RE + RE (EW)**	8	2	1	·	·	·	·
**EX**	·	1	·	·	·	·	·
**TOTAL**	101	41	22	14	3	2	8
**Percentage (%)**	52.13	21.28	11.70	7.45	1.60	1.06	4.26

**Figure 2.**
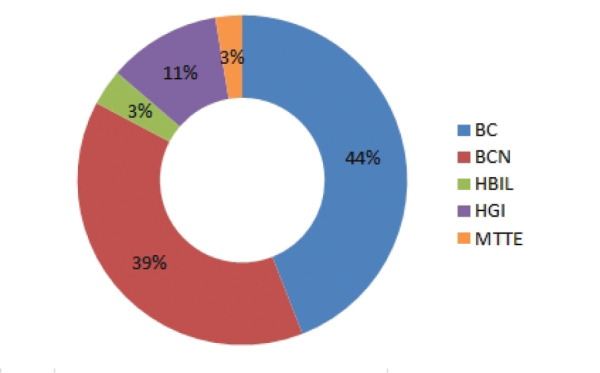
Origin herbarium of the specimens included in the dataset.

Figure 2 shows the herbarium origin of all the specimens included in this dataset and as it is expected, the number of specimens is proportional to the herbarium volume. But it is important to note that some taxa are only present in one herbarium and are missing in the rest: 20 in BCN, 18 in BC, four in HGI (*Filago lusitanica*, *Isoetes velatum*, Polygonum romanum ssp. gallicum and *Ranunculus nodiflorus*), two in HBIL (*Hesperis laciniata* and *Teucrium campanulatum*) and two in MTTE (*Atropa baetica* and *Asplenium majoricum*). These taxa are generally rare with few localities in the study area, and have been only collected once or a few times; this explains why they are only present in one herbarium, usually the closest herbarium of the taxon’s distribution. This fact highlights the importance of the small herbaria as representatives of local floras.

## Taxonomic coverage

The scientific names of this dataset are those accepted in the Catalonia Red List, which follow mainly *Flora dels Països Catalans* (Bolòs and Vigo 1984–2001) and *Flora iberica* (Castroviejo 1986–2009). The arrangement into families and orders has followed the APG III classification (APG III 2009).

**Figure 3.**
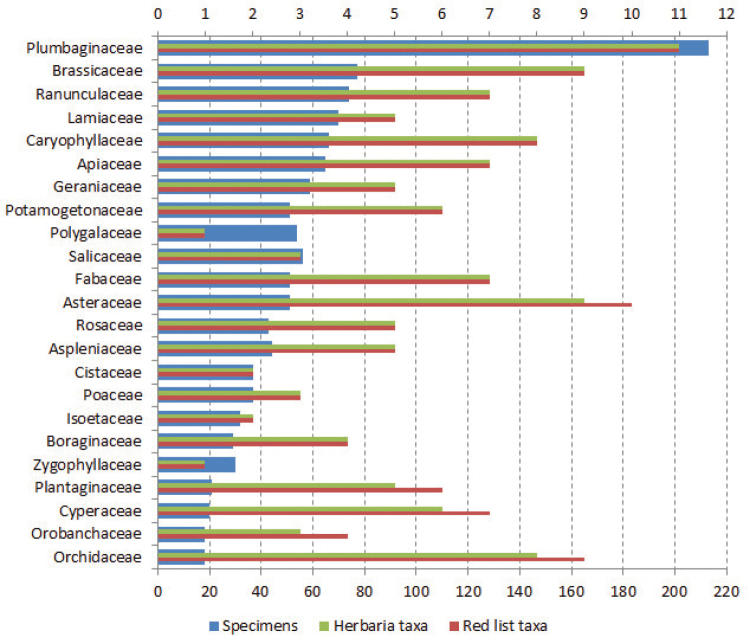
Families with 18 or more specimens (number of the taxa in the upper axis and number of specimens in the lower axis).

This dataset includes 1,618 records of threatened vascular plants (106 Pteridophyta, 6 Equisetophyta, 36 Lycopodiophyta, 11 Coniferophyta and 1,459 Magnoliophyta). Figure 3 shows the families with more specimens, that usually also have a high number of taxa (families with only three or less taxa have not been represented in the figure). The highest number of records is from Plumbaginaceae with 213 specimens; this family has also the largest number of threatened taxa with 11 species: nine *Limonium*, one *Limoniastrum* and one *Myriolepis*. It is important to note that this family has doubled the number of specimens of the second family more represented, Brassicaceae, with 77 specimens. Plumbaginaceae is a complex group with many endemisms described in the Mediterranean region with non-clear taxonomic status. Its specimens have been collected by many botanists during a large period of time, between 1866 and 2015. Almost 100 specimens have been collected by A. Curcó from 1989 to 1990, during his study of the *Limonium* genus in the Ebre river delta (Curcó 1992). Moreover, there are two species of *Limonium* among those with more than 30 specimens (Table 3). Highlight *Limonium densissimum* (57 specimens, some of them duplicates), a halophyte plant of the W-Mediterranean coast present in the Ebre river delta.

Some families are overrepresented in the dataset, like Polygalaceae with only one threatened taxon (*Polygala vayredae*) with 54 specimens (see Table 3) and Zygophyllaceae with also a single taxon (*Tetraena alba*) with 30 specimens. *Polygala vayredae* is a local endemism that occupies only an area of 12 km^2^ but it is very abundant in this area; this taxon was described in 1877 and since then many local botanists have collected it (between 1877 and 2013).

On the contrary, some families with many threatened taxa (seven or more) have few specimens (less than 50), so they are not well represented in the dataset. It is the case of Orchidaceae (nine taxa but only 18 specimens), that are usually not well represented in the herbaria due they are difficult to well press and their flowers quickly lose their color. The low presence of Cyperaceae (seven taxa but only 20 specimens) may be due to the difficulty of the identification of the *Carex* species, and the scarce presence of the threatened species in Catalonia—they only appear in a few localities in the Pyrenees. Asteraceae (10 taxa and 51 specimens) is one of the families with more threatened taxa and also the richest within plants in the Mediterranean region. The low representation of this family in the herbaria could be explained because five of the ten threatened taxa have three or less specimens; they are endemic species with a narrow distribution area (*Centaurea loscosii*, *Hieracium recoderi* or *Hieracium vinyasianum*) and species recently found in Catalonia as *Filago lusitanica* (Font and Corominas 2005) and *Galatella aragonensis* (Arrufat et al. 2008).

**Table 3.** Taxa with more than 30 specimens.

Family	Taxon	Spec.	IUCN
Geraniaceae	*Erodium sanguis-christi*	37	VU
Cistaceae	Halimium halimifolium ssp. halimifolium	34	VU
Plumbaginaceae	*Limonium bellidifolium*	39	VU
Plumbaginaceae	*Limonium densissimum*	57	VU
Brassicaceae	*Maresia nana*	40	VU
Polygalaceae	*Polygala vayredae*	54	VU
Salicaceae	*Salix tarraconensis*	47	VU
Lamiaceae	*Stachys maritima*	42	EN

The taxa with more specimens in the herbaria (Table 3) are in the most part vulnerable (VU), the UICN category with the lower threat degree, except *Stachys maritima* that is endangered (EN). This species is characteristic of the coastal sand dune vegetation and has suffered a very strong decline due to its habitat transformation; there are 24 old specimens before 1950 when the species was more or less abundant on the Catalonian coast (Barriocanal and Blanché 2002). Also *Maresia nana*, a sand dune plant and Halimium halimifolium ssp. halimifolium, a sandy substrates shrub were more or less abundant in the past as certify the 36 and 27 specimens collected before 1950 respectively. The rest of the taxa included in Table 3 are currently more or less abundant in their area, despite their threatened degree. For instance, *Salix tarraconensis*, an endemism of the Catalonian south mountains, has some populations with more than a thousand of reproductive plants and since its description in 1915 many local botanist have collected it.

The taxa with only a single specimen are shown in Table 4. All these taxa are rare and have in Catalonia only few populations in one or two localities, which explain the low representativeness in the herbaria. Only *Cochlearia glastifolia* and *Cypripedium calceolus* are more abundant than the others. *Cochlearia glastifolia* is an extinct plant in Catalonia that has been found in three localities and it has not been rediscovered since 1949, just the collection year of the BC specimen. *Cypripedium calceolus* has a wider distribution but also a high protection at regional and international level that prohibits its collection.

**Table 4.** Taxa with only one specimen. The herbarium and the collection year of each specimen are indicated.

**Family**	**Taxon**	**IUCN**	**Herb.**	**Year**
Aspleniaceae	*Asplenium majoricum*	VU	MTTE	2010
Aspleniaceae	Asplenium trichomanes ssp. inexpectans	CR	BCN	1994
Cyperaceae	*Carex diandra*	EN	BCN	1987
Cyperaceae	Carex lachenalii ssp. lachenalii	VU	BC	2007
Asteraceae	*Centaurea loscosii*	EN	BC	1917
Boraginaceae	*Cerinthe glabra*	CR	BCN	2005
Brassicaceae	*Cochlearia glastifolia*	RE	BC	1949
Orchidaceae	*Cypripedium calceolus*	VU	BCN	1986
Brassicaceae	*Hesperis laciniata*	VU	HBIL	1990
Plantaginaceae	Linaria oligantha ssp. oligantha	RE	BC	1919
Asparagaceae	*Maianthemum bifolium*	CR	BCN	1986
Orchidaceae	*Orchis cazorlensis*	CR	BC	1915
Orchidaceae	*Orchis spitzelii*	CR	BCN	1997
Ericaceae	*Phyllodoce caerulea*	VU	BCN	1995
Polygonaceae	Polygonum romanum ssp. gallicum	VU	HGI	1995
Potamogetonaceae	*Potamogeton gramineus*	CR	BC	1980
Ruppiaceae	*Ruppia drepanensis*	RE	BC	.
Saxifragaceae	*Saxifraga fragosoi*	VU	BC	1993
Amaryllidaceae	*Sternbergia colchiciflora*	CR	BCN	2005
Lamiaceae	*Teucrium campanulatum*	EN	HBIL	2009

It is important to note that some specimens have been collected at the beginning of the 21_st_ century, and in some cases they are the testimony of the first plant citation in Catalonia. It is the case of *Asplenium majoricum*, an endemism of Northern Mallorca and the Eastern Iberian Peninsula, recently founded in the south of Catalonia (Curto et al. 2012). Also *Cerinthe glabra*, a south European orophyte, was found for the first time in Catalonia in 2005 (Aymerich 2008); although Masip and Polo (1987) have mentioned some specimens in the collection of BC-Vayreda that have to be attributed to *Cerinthe major*. Likewise, the Mediterranean plant *Sternbergia colchiciflora* was found in Catalonia in 2005 for the first time (Molero et al. 2006) and in 2008 a second population has been found (Buira et al. 2009). Finally, another Mediterranean plant, *Teucrium campanulatum*, was first cited in Catalonia in 2009 (Pedrol and Conesa 2009).

There are 11 taxa of the Catalonian Red List that do not have any specimen from the study area (Catalonia) in the herbaria included (Table 5). It should be noted that, for some of them, there are specimens in those herbaria but from outside of Catalonia (either from other regions of Spain or other countries of Europe or the N of Africa), and therefore not included in this dataset. In many cases, they are plants which Catalonia represents the limit of their distribution. It is the situation of *Anthericum ramosum*, *Carex brachystachys*, *Epipogium aphyllum* and *Trapa natans*, where Catalonia is in the south limit of their distribution; *Colchicum triphyllum* and *Linaria pedunculata* in the north limit or *Pilularia globulifera* in the east limit. There are some old bibliographic cites of *Anthericum ramosum* by Vayreda (1882) but the identification of the herbarium material showed that they should be attributed to *Anthericum liliago*. This example reveals the importance of conserve voucher specimens, as they could be revised by specialists who could verify the plant identification. For some of these taxa we have found specimens in other herbaria not included in the dataset due they aren’t Catalonian herbaria or public collections. It is the case of *Pilularia globulifera* that Font et al. (1996) found it mixed with *Isoetes durieui* in a specimen collected by Sennen in 1912; this citation is the unique occurrence of this plant in Catalonia so, it is considered locally extinct. Sáez and Benito (2000) and Guardiola et al. (2013) cite some specimens of *Rhinanthus angustifolius*. The ancient presence of *Trapa natans* in Catalonia is confirmed by a Pourret’s specimen collected in 18^th^ century (Mercadal 2016).

On the other hand, *Hieracium recoderi* is an endemic taxon of Catalonia with very few localities and in GBIF there are some Catalonian specimens in other herbaria. *Woodsia pulchella* is an alpine orophyte with a single locality in the Pyrenees and there exists only some specimens (Aymerich and Sáez 2013). Finally, *Verbena supina* has been cited in Catalonia in 1902 and 1935 but any herbarium specimen has been found to support this cites.

## Taxonomic ranks

***Kingdom***: Plantae

***Phylum***: Coniferophyta, Equisetophyta, Lycopodiophyta, Magnoliophyta, Pteridophyta

***Class***: Equisetopsida, Filicopsida, Lycopodiopsida, Magnoliopsida (Monocotyledones and Dicotyledones), Pinopsida, Polypodiopsida

***Order***: Alismatales, Apiales, Asparagales, Asterales, Brassicales, Caryophyllales, Ceratophyllales, Equisetales, Ericales, Fabales, Gentianales, Geraniales, Hydropteridales, Isoetales, Lamiales, Liliales, Lycopodiales, Malpighiales, Malvales, Nymphaeales, Ophioglossales, Pinales, Poales, Polypodiales, Ranunculales, Rosales, Saxifragales, Solanales, Zygophyllales

***Family***: Alismataceae, Amaranthaceae, Amaryllidaceae, Apiaceae, Araceae, Araliaceae, Asparagaceae, Aspleniaceae, Asteraceae, Berberidaceae, Boraginaceae, Brassicaceae, Butomaceae, Caryophyllaceae, Ceratophyllaceae, Cistaceae, Convolvulaceae, Cupressaceae, Cyperaceae, Droseraceae, Dryopteridaceae, Elatinaceae, Equisetaceae, Ericaceae, Euphorbiaceae, Fabaceae, Gentianaceae, Geraniaceae, Hydrocharitaceae, Hypericaceae, Iridaceae, Isoetaceae, Juncaceae, Lamiaceae, Lentibulariaceae, Liliaceae, Lycopodiaceae, Malvaceae, Marsileaceae, Nymphaeaceae, Ophioglossaceae, Orchidaceae, Orobanchaceae, Plantaginaceae, Plumbaginaceae, Poaceae, Polygalaceae, Polygonaceae, Potamogetonaceae, Pteridaceae, Ranunculaceae, Resedaceae, Rosaceae, Rubiaceae, Ruppiaceae, Salicaceae, Saxifragaceae, Scrophulariaceae, Solanaceae, Thelypteridaceae, Thymelaeaceae, Violaceae, Woodsiaceae, Xanthorrhoeaceae, Zosteraceae, Zygophyllaceae

## Geographic coverage

The present dataset covers all the area of Catalonia (for a description of this area see “Study area” in “Project description”). The 96.48% of the records in the dataset are georeferenced. The coordinate system used is MGRS (UTM squares) and the accuracy of the grids is 10 km^2^ (the coordinates have been generalized to blur sensitive locality information due to the threatened degree of these taxa).

**Figure 4.**
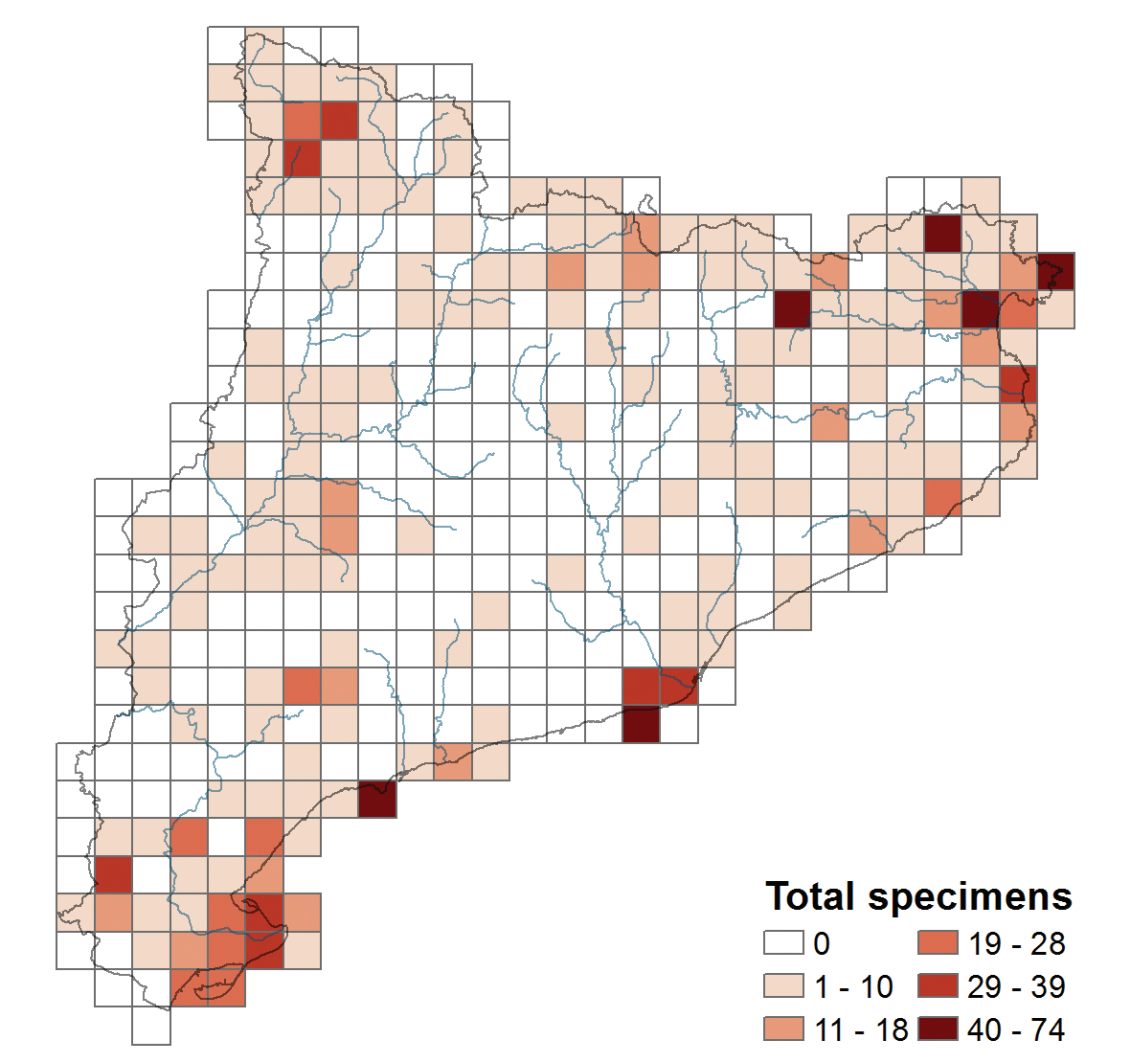
Distribution map of the number of specimens for UTM grid of 10 km^2^.

The collecting intensity map (Figure 4) permits to evaluate the regions where threatened plants have been more prospected. The areas with more than 40 specimens (the last category of the legend map) are situated in the littoral regions of Cap de Salou in the south, Delta del Llobregat in the center and Cap de Creus and Aiguamolls de l’Empordà in the north of Catalonia; and in the northeastern mountainous region of Alta Garrotxa and Serra de l’Albera.

Aiguamolls de l’Empordà is the most prospected area and also the region with the maximum number of endangered taxa (Table 6). The botanical interest of this region is high as evidence the different floristic studies done in this area during different periods (eg. Vayreda 1883, Malagarriga 1976, Farràs and Velasco 1994, Gesti 2006). The 80% of the specimens have been collected before 1925, as many of these taxa are now locally extinct in this area. Serra de l’Albera has also a large number of threatened taxa but unlike the previous region, has been visited fewer times as the 70% of the specimens were collected during the study by Font (2000). Cap de Creus is a peninsula of great floristic diversity with abrupt and rocky relief and has been visited by different botanists between 1869 and 2011. Delta del Llobregat has the 86% of the specimens collected before 1935 when this region was more natural and less urbanized than today.

On the other hand, the region of Alta Garrotxa since only two threatened taxa lives in this region has been over-collected: *Oplismenus undulatifolius* with 6 specimens and the regional endemism *Polygala vayredae*, with 51 specimens yet explained above (see Table 3).

**Table 5.** Taxa without specimens in the herbaria included in the dataset. Catalonian specimens in other herbaria are indicated.

Family	Taxon	IUCN	Other Catalonian spec.
Asparagaceae	*Anthericum ramosum*	CR	.
Cyperaceae	*Carex brachystachys*	VU	.
Colchicaceae	*Colchicum triphyllum*	RE	.
Orchidaceae	*Epipogium aphyllum*	CR	.
Asteraceae	*Hieracium recoderi*	CR	MA-553699
			P-04302573 (holotype)
			VAL-85707
			VAL-85735
			VAL-75322
			VAL-24946
			L. Sáez, herb. pers.
Plantaginaceae	*Linaria pedunculata*	RE	.
Marsileaceae	*Pilularia globulifera*	RE	MA-2360
Orobanchaceae	*Rhinanthus angustifolius*	VU	L. Sáez, herb. pers. (4 specimens)
Lythraceae	*Trapa natans*	RE	MAF-POURRET-770
Verbenaceae	*Verbena supina*	RE	.
Woodsiaceae	*Woodsia pulchella*	CR	MAF-130204
			MAF-130205
			L. Sáez, herb. pers.

**Table 6.** Number of specimens and taxa of the most prospected areas, with more than 40 specimens.

UTM (regions)	Dataset		Catalonia Red Book
	Spec.	Taxa	
31TEG07 (Aiguamolls de l’Empordà)	72	16	19
31TEG28 (Cap de Creus)	59	10	7
31TDG57 (Alta Garrotxa)	57	2	2
31TDG99 (Serra de l’Albera)	58	12	13
31TDF16 (Delta del Llobregat)	52	6	7
31TCF44 (Cap de Salou)	47	8	7

**Figure 5.**
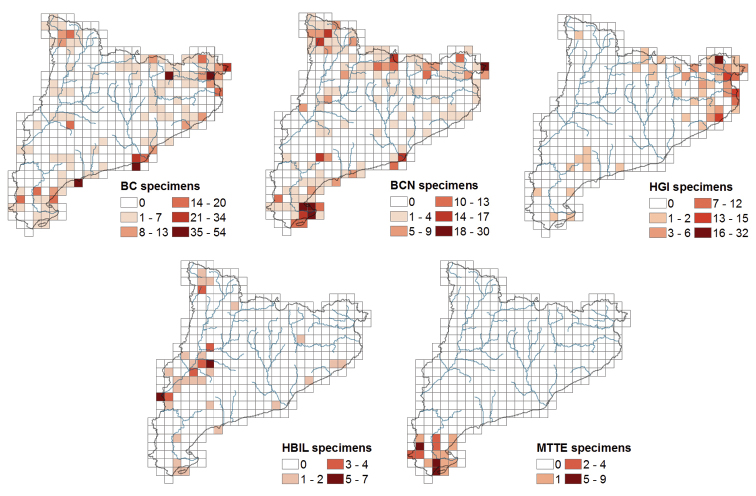
Distribution map of the specimens for UTM grid of 10 km^2^ for each herbarium.

If we evaluate the collecting intensity map separated for each herbarium (Figure 5) we can see that local herbaria like HGI, HBIL and MTTE host a good representation of specimens from its surrounding area. In the case of the biggest herbaria (BC), the regions most prospected match those indicated in Table 6: Aiguamolls de l’Empordà, Delta del Llobregat, Cap de Salou and Alta Garrotxa. However, in BCN herbarium the most prospected area is Delta de l’Ebre, with 100 specimens collected during the floristic study of this region by Curcó (2003), although there are also a high number of specimens from Cap de Creus collected during the studies of Franquesa (1995) and Sáez (1997).

Table 7 shows the number of specimens and taxa of hotspot areas (with elevated number of threatened taxa) designated as Important Plant Areas (IPA) in the Red Book of Catalonia. In fact, all they have different protect regulation included in the Plan for Spaces of Natural Interest (PEIN, DOGC 1992) except the region of Empúries-l’Armentera situated in the littoral, with any protection directive. The most restricted protection is for Aigüestortes, situated in the east of the Catalonian Pyrenees and catalogued as National Park in 1955, so legal permits are needed to collect plants. But in fact, only 22% of the specimens have been collected before this regulation and the 69% have been collected between 1978 and 2009 by the University of Barcelona botanist team that studied this region (Carrillo and Ninot 1992, Guardiola et al. 2009).

**Table 7.** Number of specimens and taxa of the hotspot areas defined in the Red Book of Catalonia; only squares with more than 8 taxa are recorded.

UTM	Catalonia Red Book	Dataset	
		Spec.	Taxa
31TEG07 (Aiguamolls de l’Empordà)	19	72	16
31TEG15 (Montgrí - baix Ter)	13	35	13
31TDG99 (Serra de l’Albera)	13	58	12
31TEG06 (Empúries - l’Armentera)	12	14	10
31TCF00 (Delta de l’Ebre)	12	19	6
31TCH32 (Naut Aran)	11	33	8
31TCH21 (Aigüestortes)	11	32	7
31TEG17 (Cap de Creus)	10	20	11
31TBF72 (Massís del Port)	10	37	10
31TBE99 (Delta de l’Ebre)	10	24	9
31TCE09 (Delta de l’Ebre)	10	21	4
31TCH22 (Naut Aran)	9	22	7
31TDF27 (Delta del Llobregat)	9	35	9
31TBF90 (Delta de l’Ebre)	9	17	7
31TCF55 (Tarragona)	9	10	5
31TBF71 (Massís del Port)	9	11	5
31TCG31 (Ivars d’Urgell)	8	17	6

Another important region is Ivars d’Urgell (31TCG31), a small area of halophilous and gypsum vegetation with 8 threatened taxa and included in the PEIN; this region is represented in the herbaria by 6 taxa and 17 specimens.

On the other hand, some UTM squares with a high number of threatened taxa have a poor presence in the herbaria. In some cases because they are littoral squares with a large part of the area in the sea like 31TBE99 and 31TCE09 from Delta de l’Ebre and 31TEG17 from Cap de Creus. But in other, the prospecting intensity is too low relating the number of threatened taxa; for instance, 31TCF55 in the littoral of Tarragona with only 10 specimens collected between 1893 and 1993 and 31TBF71 in the mountainous Massís del Port in the south with 11 specimens collected between 1917 and 2008.

**Figure 6.**
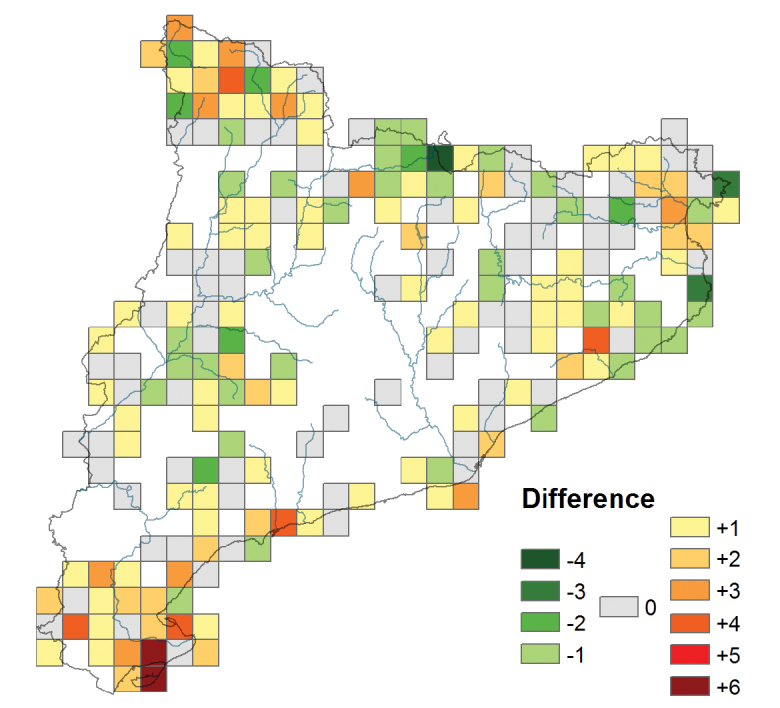
Comparative map of the number of taxa of the dataset and that according to Sáez et al. (2010: 733). Gray color indicates that all the taxa cited in this book have a specimen in the herbaria studied; yellow-red color indicates that the dataset doesn’t include all the taxa published in this book and green color indicates that there are more taxa in the dataset that those published in this book.

In Tables 6 and 7 we can see that in some cases the number of taxa included in the dataset for each UTM square is lower or higher than the one of the Red Book, Figure 6 shows these differences. In the 34.26% of the squares the difference is zero (grey color), indicating that all the taxa cited in this book have minimum a specimen in the herbaria studied. The 45.37% of squares have a positive difference (colored yellow to red), meaning that the dataset doesn’t include all the taxa published in this book. The regions less representatives in the herbaria (red squares) are in the south: two squares in Delta de l’Ebre yet indicated in Table 7.

The 20.37% of the squares have a negative difference (green color), indicating that there are more taxa in the dataset than in the Red Book. This is due, on one hand, to new citations published by different local botanists after the Red Book publication in 2010. For instance, in the UTM square 31TDG19, situated in La Cerdanya in the center of the Catalonian Pyrenees, the dataset includes specimens of *Gagea pratensis* and *Gagea reverchonii* published by Aymerich (2013) and so not indicated in the Red Book published three years before. This fact shows that herbaria are dynamic libraries of taxa if we compare with books that may become obsoletes once published. On the other hand this negative difference may also be due to some specimens that can provide new citations and are still to be studied and published. This second fact demonstrates that herbaria are important sources of hidden data.

Finally, highlight that for more of the 50% of the squares (difference zero or negative) all the taxa have almost a specimen which indicates the good representativeness of this kind of flora in the herbaria.

## Temporal coverage

**Figure 7.**
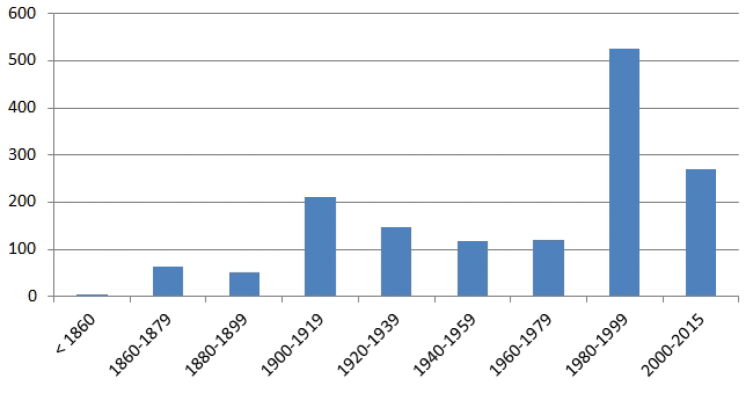
Collecting years according the specimens labels.

The 92.27% of the specimens have the collecting year indicated in the label. Among this, the temporal coverage is between 1861 and 2015 (Figure 7). There are 6 specimens from the Salvador’s collection conserved in the BC herbarium, collected between 17_th_ and 18_th_ century (Ibáñez 2006), but without specific year in the label (in the figure are indicated as before 1860). In some cases, these old specimens are the testimony of the presence of a taxon in an extinct locality as the specimens of *Hydrocotyle vulgaris* and *Stachys maritima* from the coast of Barcelona.

The maximum number of specimens was collected since 1980 when the number of botanists dedicated to floristic studies significantly increases. After the regulation of the threatened flora in Catalonia with the publication in 2008 of the Catalogue of Endangered Flora (DOGC 2008) the specimens collected have been declined and there are only 112 specimens between 2009 and 2015 when legal permits are needed (the 6.92% of the whole dataset).

**Figure 8.**
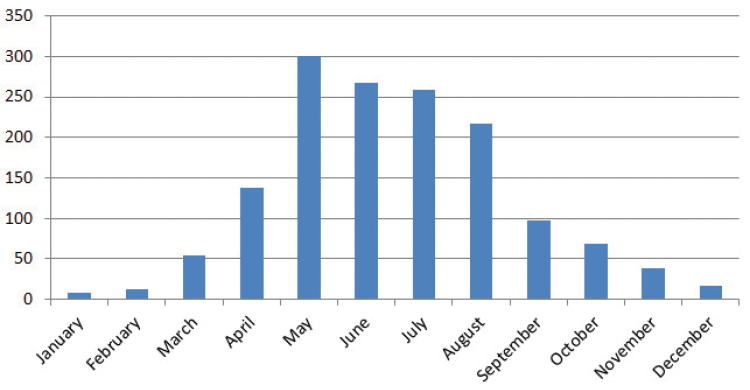
Collecting months according the specimens labels.

On the other hand, Figure 8 shows that the most part of specimens have been collected in spring and summer, when almost all the taxa are in flowering state in the study area.

## Methods

### Step description

The creation of this dataset has included different processes: (1) specimens searching, (2) specimens digitalization, (3) dataset documentation, (4) dataset unification and analysis and (5) dataset publication at the GBIF portal. The first three steps have been done by the responsibility of the curator of each herbarium.

1. The search of all the specimens of the Catalonian threatened taxa has been done exhaustively thanks to a list of synonyms prepared in Nualart et al. (2012) since specimens may be stored in the herbaria under different synonymous names.

2. The digitalization of the specimens has been done by different programs that allow the inclusion in the database of all the information in the label. In BC herbarium this step is managed with Herbar (Pando et al. 1994-2010), in BCN with an own application developed in Access, in HGI with an own program developed in File Maker Pro 2.0 by Macintosh (Campos et al. 1995) and in MTTE with MuseumPlus by ZetCom (http://www.zetcom.com/en/products/museumplus/)(the program used by all the Museums in Catalonia).

3. The dataset documentation includes all the revisions that have been taken place to improve the information of each specimen, such as check the locality of collecting thanks to information from the botanists’ documentation and the database. Also the names of the collectors have been checked—when they were not clear—according to the calligraphy in the label. Moreover, all the localities have been geo-referenced wherever possible using coordinates UTM 10 Km^2^ (MGRS system) from Catalonia geographical viewer (http://www.icc.cat/vissir3). In those specimens with more precise coordinates in the label, the coordinates have been generalized to blur sensitive information due to the threatened degree of these taxa. Furthermore, the locality information has been completed indicating wherever possible, the province and the municipality according to ICC (2009). Finally, the indication of the country and province has been standardized following the ISO 3166.

4. For the dataset unification a list of fields has been decided considering the maximum possible number of common fields in the different herbaria databases. This list includes the following information: (1) the catalog number, (2) the taxon name, (3) the information about the identification (date and researcher), (4) the locality information (country, province, municipality, locality name, UTM coordinates and altitude in meters), (5) the ecology and (6) the gathering information (date, collector, collector number and exsiccate or field campaign). Each curator has prepared its dataset in an Excel table and finally all the records of each herbarium have been unified in a single dataset. The analysis for describe the dataset (tables and graphics presented in this paper) have been carried out in Excel from this unique dataset. Distribution maps have been created using ArcGis 10.2.

5. For the publication in the GBIF portal all the data have been accommodated to fulfil the Darwin Core Standard (Wieczorek et al. 2012). The Darwin Test (Ortega-Maqueda and Pando 2008) has been used to convert coordinates from UTM to decimal degrees which are used in the Darwin Core format. The Integrated Publishing Toolkit (IPT v2.0.5) of the GBIF.es (http://www.gbif.es:8080/ipt) has been used to upload the Darwin Core Archive and to fill out the metadata.

### Quality control description

Once the dataset has been completed (after the forth step of the methodology) a revision of the data has been carried out by comparing the distribution map obtained from the herbarium data of each taxon with that published in the Red Book and in the “Biodiversity data bank of Catalonia” (Font 2016). The specimens’ observations not recorded in these published distribution maps have been subjected to an accurate revision to ensure its validity. In these cases, the geospatial information has been checked and herbarium specimens have been reviewed to confirm taxonomic identification. This process has enabled to debug data and remove those specimens not well identified.

Other processes of quality control have been implemented in the third step yet explained in the methodology.

## Dataset description

**Object name**: Darwin Core Archive Threatened plants of Catalonia.

**Character encoding**: UTF-8

**Format name**: Darwin Core Archive format

**Format version**: 1.0

**Distribution**: http://www.gbif.es/ipt/resource?r=threatenedCAT

**Licenses of use**: This Dataset is made available under the Open Data Commons Attribution License: http://www.opendatacommons.org/licenses/by/1.0

**Metadata language**: English

**Date of metadata creation**: 2016-19-12

**Hierarchy level**: Dataset
